# Feature Matching Optimization of Multimedia Remote Sensing Images Based on Multiscale Edge Extraction

**DOI:** 10.1155/2022/1764507

**Published:** 2022-06-02

**Authors:** Yani Wang, Jinfang Dong, Bo Wang

**Affiliations:** ^1^Xi'an University, Xi'an, Shaanxi 710000, China; ^2^Shaanxi Meteorological Service Center of Agricultural Remote Sensing and Economic Crops, Baoji, Shaanxi 721199, China; ^3^Shaanxi Geomatics Center, Ministry of Natural Resources, Xi'an, Shaanxi 710054, China

## Abstract

In order to solve the problem of low efficiency of image feature matching in traditional remote sensing image database, this paper proposes the feature matching optimization of multimedia remote sensing images based on multiscale edge extraction, expounds the basic theory of multiscale edge, and then registers multimedia remote sensing images based on the selection of optimal control points. In this paper, 100 remote sensing images with a size of 3619*∗*825 with a resolution of 30 m are selected as experimental data. The computer is configured with 2.9 ghz CPU, 16 g memory, and i7 processor. The research mainly includes two parts: image matching efficiency analysis of multiscale model; matching accuracy analysis of multiscale model and formulation of model parameters. The results show that when the amount of image data is large, feature matching takes more time. With the increase of sampling rate, the amount of image data decreases rapidly, and the feature matching time also shortens rapidly, which provides a theoretical basis for the multiscale model to improve the matching efficiency. The data size is the same, 3619 × 1825, which makes the matching time between images have little difference. Therefore, the matching time increases linearly with the increase of the number of images in the database. When the amount of image data in the database is large, a higher number of layers should be used; when the amount of image data in the database is small, the number of layers of the model should be reduced to ensure the accuracy of matching. The availability of the proposed method is proved.

## 1. Introduction

**Figure 1:**
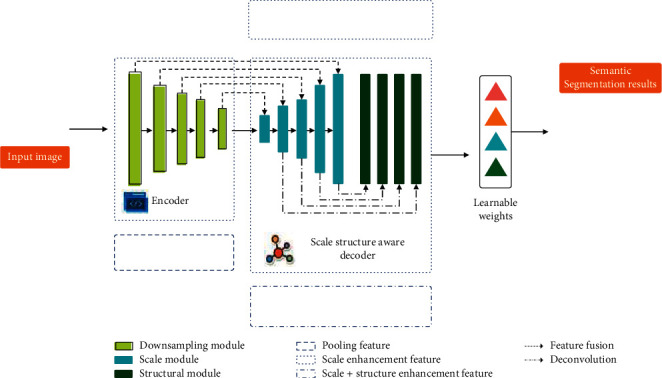
Multiscale image feature information extraction and flow based on weight learning.

With the rapid development of remote sensing technology, aerial photography, UAV, and vehicle mobile measurement system, it is possible to obtain various image data reflecting the characteristics of natural and human activities quickly, dynamically, and on a large scale [1]. Images contain rich information and have the advantages of intuition, image, and easy understanding. They play an extremely important role in human perception of the external world [2]. In reality, people extract the physical characteristics and spatial information of various target objects in the objective world through images to study the spatial position, shape, attribute, change, and relationship with the surrounding environment. Therefore, the research on image processing technology has always been the most important research content in the field of photogrammetry, remote sensing, and computer vision. Due to the influence of a series of factors such as atmospheric refraction, terrain fluctuation, and the change of internal and external orientation elements of the sensor, the quality of the obtained image is reduced or there is a lack of useful information, which brings difficulties to image processing and target recognition. It is difficult to rely on vision-based processing only. The multiscale analysis method is an effective means [3]. Through the decomposition of the image on different scales by multiscale technology, the image feature information can be expressed in different degrees at different scales, which is conducive to a better understanding of the details of the image, fully extract the image feature information, and obtain ideal results in the process of image matching and target recognition. The multiscale image feature information extraction and process based on weight learning is shown in Figure 1 [4].

## 2. Literature Review

To solve this research problem, Ren et al. proposed a method of extracting point features by a gray method [5]. Lv et al. proposed a new operator Forstner operator based on the least square principle and measured by the point gray error ellipse [6]; Queiroz et al. proposed sift operator, i.e. variable ratio invariant feature point [8]; Yang et al. improved Moravec operator with the same idea and proposed Plessey corner detection operator [9]. Dongand Lin compared Harris, Cottier, Forstner, and other operators, and considered that the effect of Forstner operator was second only to Harris operator [10]. Xu et al. compared Plessey, Forstner, and susan-2d operators and concluded that Forstner operator achieved the best results in the comprehensive comparison of clarity, invariance, stability, uniqueness, and interpretability. However, in the application, it is found that Harris corner is very sensitive to the change of image scale. When the image size is inconsistent, Harris corner is not a good result. Basically, all point feature extraction operators have such problems [11]. Chen et al. used differential geometry to extract linear targets in images, including lines and curves [12]. Ma et al. combined with the least square and Kalman filter, used the gray section perpendicular to the road direction for road tracking. Due to the different manifestations of the same target in different resolution images, multiresolution analysis can combine the advantages of the two to obtain better recognition results [13]. Yao et al. analyzed the application of multiscale analysis theory in road extraction in detail and gave a theoretical framework, which has guiding significance for road extraction based on multiscale analysis [14].

## 3. Method

### 3.1. Basic Theory of Multiscale Edge

Wavelet analysis is born after Fourier analysis. Wavelet bases are considered to be sparse when representing singular points, but it is not suitable for representing line singular targets. Therefore, it is difficult to use wavelet basis to represent edge information. Forcibly using wavelet basis to describe edge linear targets will bring ringing phenomenon to the experimental results of image denoising [15]. Donoho proposed the edge transform when studying the restoration of noisy image data. The weldlet transform can approximately optimally describe the “horizontal model.” Donoho found that the weldlet decomposition based on the cost function can achieve minimax risk estimation. When representing an image with a large number of linear edges, the weldlet distortion rate is very small, and the optimal approximation can be achieved nearly [16].

### 3.2. Multimedia Remote Sensing Image Registration Based on Optimal Control Point Selection

#### 3.2.1. Solving the Optimal Solution of Projection Variation Based on Least Square Method

We use the projection transform *H* (topography) to describe the matching relationship between two image control point pairs. Let *P*_*t*_(*x*, *y*) and *P*_*r*_(*u*, *v*) represent the coordinates of a pair of matching control point pairs of test images *C* and *D*, respectively [17]. According to the projection transformation relationship:

(1) $$\begin{array}{c} u=\frac{h_{1}x+h_{2}y+h_{3}}{h_{7}x+h_{8}y+1},\\ u=\frac{h_{4}x+h_{5}y+h_{6}}{h_{7}x+h_{8}y+1}. \end{array}$$

In order to obtain the projection transformation matrix *H*=[*h*_1_, *h*_2_, *h*_3_, *h*_4_, *h*_5_, *h*_6_, *h*_7_, *h*_8_], at least 4 pairs of matching control point pairs are required on *I*_*t*_ and *I*_*r*_ to solve 8 parameters. Considering that there is matching error in the control point pairs distributed on *I*_*t*_ and *I*_*r*_ in practical engineering application, if 4 pairs of matching control point pairs are directly used for calculation, large matching error will be introduced. Therefore, this method uses multipair (greater than 4) control point matching to approximately calculate the projection transformation parameters of *I*_*t*_ and *I*_*r*_ through the least square method. Let *P*_*ti*_(*x*_*i*_, *y*_*i*_), *i*=1,2,3,…, *N* represent the coordinates of *I*_*t*_ control points, and *P*_*ri*_(*u*_*i*_, *v*_*i*_), *i*=1,2,3,…, *N* represent the coordinates of control points matching *P*_*ti*_(*x*_*i*_, *y*_*i*_) in *I*_*r*_ [18].

When *n* is determined, the *H* ideal approximate solution is obtained, and the average distance between the control points of the two images is the smallest. *N* is the number of input control point pairs. According to the idea of the least square method proposed in this chapter, the minimum value of *n* is 4. Experiments show that when *n* is 6 or 7, this algorithm can achieve a good compromise in registration accuracy and efficiency. The control point of *I*_*t*_ is mapped to *I*_*r*_ through projection transformation, and the average distance from *I*_*r*_ control point is

(2) $$\begin{array}{c} d_{t2r}=\sqrt{\frac{1}{N}\sum_{i=1}^{N}{\left|H\left(x_{i},y_{i}\right)-\left(u_{i},v_{i}\right)\right|}^{2}}. \end{array}$$

According to the inverse matrix *H*^−1^, the control points of *I*_*r*_ are mapped to *I*_*t*_ through projection transformation, and the average distance from *I*_*t*_ control points is

(3) $$\begin{array}{c} d_{t2r}=\sqrt{\frac{1}{N}\sum_{i=1}^{N}{\left|\left(x_{i},y_{i}\right)-H^{-1}\left(u_{i},v_{i}\right)\right|}^{2}}. \end{array}$$

#### 3.2.2. Selection and Correction of Control Point Pairs

The selection of control point pairs and automatic matching are two key points of projection transformation. Firstly, the algorithm inputs the control point pair of the test image and the reference image according to the human visual and image features, which can ensure that the distribution position and area of the feature points selected on the test image and the reference image are relatively consistent and evenly distributed in the image so as to lay a good foundation for establishing the accurate matching of the control point pair.

**Figure 2:**
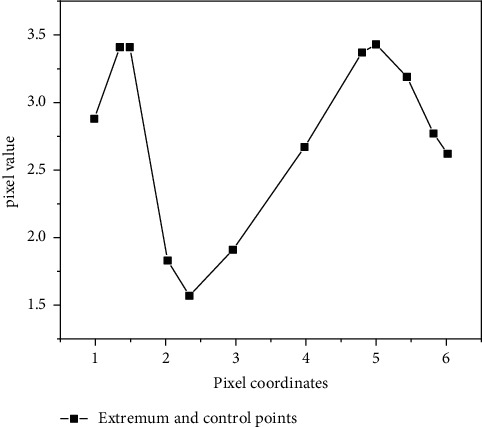
Difference between input control points and image extreme points.

Since the control points input manually for the first time are not necessarily the real extreme points of the image, as shown in Figure 2, the control points of the test image need to be corrected to distribute them to the extreme points of the image so as to enhance the matching stability and improve the antinoise ability [19]. In this algorithm, the Taylor expansion of Gaussian difference function (dog) is used to find the extreme points near the control points of the test image through linear interpolation, so that the input control points are distributed on the extreme points with more stable and higher position accuracy.

Set point a as the known input control point, use point a to estimate the value of a nearby extreme point *B*, and set *X*=(*x*, *y*, *σ*)^*T*^ as the offset of point *B* relative to point a. According to the Taylor function expansion of dog function (fitting function):

(4) $$\begin{array}{c} D\left(X\right)=D+\frac{\partial D^{T}}{\partial X}X+\frac{1}{2}X^{T}\frac{\partial^{2}X}{\partial X^{2}}. \end{array}$$

By deriving and making (4) equal to zero, the offset of the extreme point can be obtained as follows:

(5) $$\begin{array}{c} \hat{X}=-\frac{\partial^{2}D^{\partial 1}}{\partial X^{2}}\frac{\partial D}{\partial X}. \end{array}$$

When the offset of $\hat{X}$ in any dimension is greater than 0.5, it indicates that the extreme point is closer to the adjacent point *C* of *A*. Update point a to point *C* and continue the iteration until the offset of any dimension of $\hat{X}$ is less than 0.5, and the iteration process ends. Add $\hat{X}$ to the current point to obtain the exact position of the control point [20].

#### 3.2.3. Matching of Control Point Pairs

According to equations (2) and (3), the initial matching errors *d*_*t*2*r*_ and *d*_*r*2*t*_ of the control point pair can be calculated. The initial values *d*_*r*2*t*_ and *d*_*t*2*r*_ need to be further reduced by adjusting the control points [21]. Because the control point of the test image is close to the ideal extreme point after being processed by dog operator, this algorithm mainly adjusts the position of the corresponding control point of the reference image. According to the marking sequence of the control point pair, it automatically moves according to the set step *s* in the up, down, left, and right directions, respectively, and searches the best matching position of the reference image control point based on the principle of obtaining the minimum value with *d*_*t*2*r*_ and *d*_*r*2*t*_ [22]. Suppose *n* pairs of control points are input in the test image and reference image, and *N* takes 6 or 7 in the experiment. The control points of the test image are corrected by the dog operator, and the automatic search algorithm of the matching control points corresponding to the reference image is: calculate the initial error: calculate the matching errors *d*_*t*2*r*_ and *d*_*r*2*t*_ of the initial control points according to (2); iterative adjustment of reference image control points: the positions of other control points are fixed. For the *i*th control point, move the coordinates according to step *S*. When *d*_*t*2*r*_ and *d*_*r*2*t*_ are no longer reduced, the position of the control point at this time is its best position, and then adjust the position of the next control point in turn [23]; iteration abort: when the matching distance error of the control point pair of the test image and the reference image is less than a preset threshold, the iteration aborts and the algorithm ends.

(6) $$\begin{array}{c} \sqrt{\frac{1}{N}\sum_{i=1}^{N}{\left|H\left(x_{i},y_{i}\right)-\left(u_{i},v_{i}\right)\right|}^{2}}<T_{h},\\ \sqrt{\frac{1}{N}\sum_{i=1}^{N}{\left|\left(x_{i},y_{i}\right)-\left(u_{i},v_{i}\right)\right|}^{2}}<T_{h}. \end{array}$$

How to define *T*_*h*_ is a very important problem. The definition of *T*_*h*_ is too small, the matching control point pair will deviate from the actual feature points of the image, and the ideal registration effect cannot be achieved; too much definition of *T*_*h*_ will lead to large registration error. Through experimental analysis, it is found that when *T*_*h*_ is distributed between (0.3, 0.5), the image registration effect is ideal [24].

#### 3.2.4. Algorithm Implementation Process

In order to solve the problem of registration accuracy of different modal remote sensing images, this chapter proposes a multimodal image registration algorithm based on the selection of optimal matching points. The flow of the algorithm is as follows:

Initialize the algorithm, set the iteration end threshold parameter *T*_*h*_, and adjust the step size *S*; manually input *n* pairs of control points in test image *I*_*t*_ and reference image *I*_*r*_:

(7) $$\begin{array}{c} P_{t}^{1},P_{t}^{2},P_{t}^{3},P_{t}^{4},\ldots P_{t}^{N},P_{r}^{1},P_{r}^{2},P_{r}^{3},P_{r}^{4},\ldots P_{r}^{N}. \end{array}$$

The dog operator is used to adjust the control point of the test image so that it is located at the extreme point to obtain a stable and accurate control point; using projection transformation, *H* and *H*^−1^ are calculated based on the coordinates of control point pairs, and *H* and *H*^−1^ are substituted into equations (2) and (3) to obtain the initial matching errors *d*_*t*2*r*_ and *d*_*r*2*t*_ of control point pairs; for *I*_*r*_, the position of control point is automatically adjusted point by point, the adjustment step is *s*, and *d*_*r*2*t*_ and *d*_*t*2*r*_ are recalculated; when *d*_*t*2*r*_ < *T*_*h*_ and *d*_*r*2*t*_ < *T*_*h*_, the automatic adjustment process ends; calculate *h* based on the position coordinates of the control point pair; using H, traverse all pixels of *I*_*t*_, construct and project to image *I*_*r*_, and the registration algorithm ends [25].

## 4. Results and Analysis

In this paper, 100 remote sensing images with a size of 3619*∗*825 with a resolution of 30 m are selected as experimental data. The computer is configured with 2.9 Ghz CPU, 16 g memory, and i7 processor. The research mainly includes two parts: image matching efficiency analysis of multiscale model; matching accuracy analysis of the multiscale model and formulation of model parameters.

### 4.1. Image Matching Efficiency Analysis of Multiscale Model

**Figure 3:**
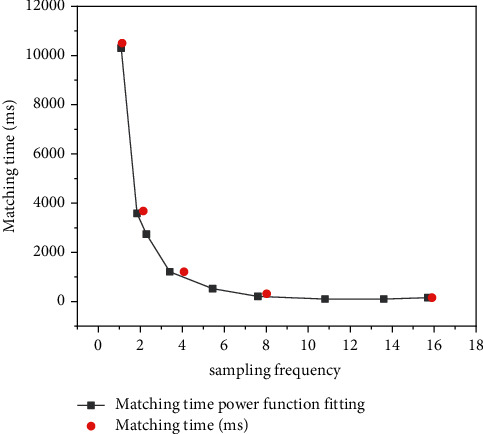
Variation trend of matching time with sampling rate.

1. Correlation between matching rate and image sampling rate select two images for matching calculation, one as the image to be matched and the other as the target image, and resample the image to be matched to varying degrees.  Table 1 shows the variation of image matching time with sampling rate.
2. Figure 3 shows the change of image matching time with sampling rate. It can be seen from the figure that with the increase of sampling rate, the image matching time first decreases rapidly, and then changes gradually gently. This change law shows that when the amount of image data is large, the feature matching takes more time. With the increase of sampling rate, the amount of image data decreases rapidly, and the feature matching time also shortens rapidly. This provides a theoretical basis for the multiscale model to improve the matching efficiency.
3. Matching efficiency between multiscale model and single-layer image database

**Figure 4:**
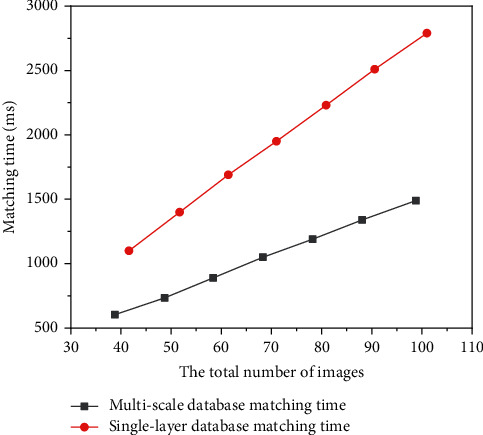
Linear fitting of matching time of different methods.

As compared in order to further compare and analyze the difference of feature matching efficiency between multiscale model and traditional single-layer image database, five groups of image databases are created, and the total number of images in the database are 40, 50, 60, 70, 80, 90, and 100, respectively. Multiscale models are built for 7 groups of databases. The feature matching time of 7 groups of image databases under different methods is shown in Table 2.  Figure 4 shows the efficiency difference of feature matching under different methods. It can be found that the matching time fitting equation has linear characteristics: the reason why the matching time is linearly related to the total number of images is that the remote sensing images selected in the experiment have the same resolution and the data size is the same, which is 3619 × 1825, which makes the matching time between images have little difference. Therefore, the matching time increases linearly with the increase of the number of images in the database. In practical application, the image size is different. Therefore, in practical application, there is not necessarily a good linear relationship between the matching time and the total number of images.

When the total number of images is the same, the feature matching time of the multiscale model is always less than that of single-layer database, and the advantages of the multiscale model are more obvious with the increase of the total number of images. Therefore, with the increase of the total number of images, the difference of matching efficiency between the two methods increases gradually. In practical application, the database usually contains hundreds of remote sensing images. At this time, the matching efficiency of the multiscale model will be much higher than that of single-layer image database.

### 4.2. Image Matching Accuracy of Multiscale Model

In this paper, the matching accuracy of the multiscale model is studied by changing the image size. The process is as follows: (1) 100 images are divided into 5 groups, 20 images in each group, of which the image size in the group is the same and the image size between the groups is different; (2) each image is divided into five layers, and the multiscale model is used for feature matching; (3) record the current number of layers in each group where the real matching image is wrongly eliminated, reduce the number of layers, until the real matching image obtains the accurate matching position, and record the best number of layers with the highest final matching accuracy [26]. The experimental results of 5 groups of data are shown in Table 3. It can be found from Table 3 that with the decrease of image size, the optimal number of layers also decreases, and when the image size is reduced to 226 × 114, the correct matching results cannot be obtained by using multi-scale model for feature matching. This is because when the amount of image data is small, the total number of feature points extracted from the image is small, and a small number of feature points cannot fully ensure the matching accuracy of the image. Therefore, when building a multiscale model for the image database in practical application, the amount of image data at the highest level should be greater than 226 × 114.

**Figure 5:**
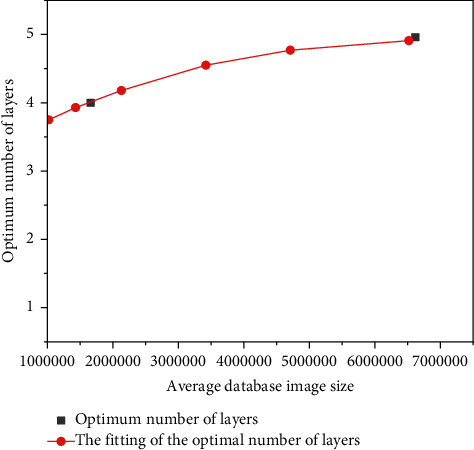
Functional relationship between image data volume and optimal layering number.

The data in Table 3 are linearly fitted to obtain the functional relationship between the average data volume of the database image and the optimal number of layers. As shown in Figure 5, it can be found that when the amount of image data in the database is large, a higher number of layers should be adopted; when the amount of image data in the database is small, the number of layers of the model should be reduced to ensure the accuracy of matching. Using the logarithmic function equation of database image and the optimal number of layers, the optimal number of layers of image database in practical application can be obtained so as to ensure the feature matching accuracy of database image.

## 5. Conclusion

In this paper, a feature matching optimization of multimedia remote sensing images based on multiscale edge extraction is proposed. The proposed algorithm can not only efficiently complete the feature point matching operation between images but also accurately screen the best matching images from the database; with the increase of the total number of images in the database, the advantages of this method are more obvious. This research will provide the possibility for efficient, real-time, and dynamic matching of remote sensing image database. The multiscale method also has defects, because the local features of the image exist in a certain scale range, so a feature point may have several different feature scales at the same time, which increases the difficulty of subsequent matching. In the future, it is necessary to find a method to make the local features represented by representative feature points.

## Figures and Tables

**Table 1:** Variation of the first image matching time with the sampling rate.

Sampling rate	1	2	4	8	16

Image size	3619 × 1825	1810 × 912	905 × 456	452 × 228	226 × 114
Total number of feature points	39624	32904	11058	3077	761
Matching time/ms	10452	3711	1051	281	69

**Table 2:** Comparison of matching efficiency between the new method and single-layer database.

Total number of images	40	50	60	70	80	90	100

Single-layer database matching time/s	1131	1414	1697	1980	2263	2546	2829
Multiscale database matching time/s	601	752	903	1053	1204	1355	1505

**Table 3:** Matching accuracy of different image sizes.

Image size	3619 × 1825	1810 × 912	905 × 456	452 × 228	226 × 114

Total number of grids	6604675	1650720	412680	103056	25764
False rejection layers	Nothing	5	4	3	2
Optimal number of layers	5	4	3	2	1

## Data Availability

The data used to support the findings of this study are available from the corresponding author upon request.
